# Effects of Marine Sand on the Microbial Degradation of Biodegradable Plastics in Seawater and Biofilm Communities that Formed on Plastic Surfaces

**DOI:** 10.1264/jsme2.ME22047

**Published:** 2022-10-15

**Authors:** Tomohiro Morohoshi, Asuka Taniguchi, Ami Sugawara, Tomohiro Suzuki, Shunsuke Sato

**Affiliations:** 1 Graduate School of Regional Development and Creativity, Utsunomiya University, 7–1–2 Yoto, Utsunomiya, Tochigi 321–8585, Japan; 2 Center for Bioscience Research and Education, Utsunomiya University, 350 Mine-machi, Utsunomiya, Tochigi 321–8505, Japan; 3 Agri-Bio & Supplement Research Laboratories, Kaneka Corporation, 1–8 Miyamae-cho, Takasago-cho, Takasago, Hyogo 676–8688, Japan

**Keywords:** biodegradable plastic, poly(3-hydroxybutyrate-*co*-3-hydroxyhexanoate), biofilm, marine sand, seawater

## Abstract

Four types of biodegradable plastics were evaluated for their biodegradability in seawater collected at Ajigaura coast, Japan, in the presence or absence of marine sand. One of the plastics, poly(3-hydroxybutyrate-*co*-3-hydroxyhexanoate) (PHBH), showed a degree of biodegradation in a seawater sample, and the addition of marine sand markedly accelerated its biodegradation. The addition of marine sand did not affect the bacterial composition of the biofilm that formed on PHBH, and the family *Rhodobacteraceae*, which was predicted to contribute to the degradation of PHBH, was dominant in biofilm communities regardless of the addition of marine sand. Marine sand may serve as a bacterial source, resulting in the accelerated degradation of PHBH.

Plastic pollution has become a social issue with devastating effects on aquatic environments (Jambeck *et al.*, 2015). The use of biodegradable plastics is one solution for preventing the generation of microplastics in aquatic environments. Biodegradable plastics are degraded into carbon dioxide (CO_2_) by various microorganisms (Tokiwa and Calabia, 2007). Many types of biodegradable plastics have been developed and used to prevent environmental pollution caused by waste plastics (Tokiwa and Calabia, 2007). Poly(butylene succinate-*co*-butylene adipate) (PBSA) and poly(butylene adipate-*co*-terephthalate) (PBAT) are oil-based biodegradable plastics with ester bonds that are degraded by enzymes secreted by microorganisms (Jiang *et al.*, 2006; Tokiwa and Calabia, 2007). Poly(lactic acid) (PLA) and polyhydroxyalkanoates (PHAs) are bio-based biodegradable plastics derived from renewable resources (Tokiwa and Calabia, 2007). These plastics are degraded by the formation of biofilms composed of various bacteria with biodegradable plastic-degrading activities. A biofilm is an assembly of surface-associated microbial cells that are enclosed in an extracellular matrix (Donlan, 2002). We previously examined the degradability of biodegradable plastic films in seawater and freshwater samples. One PHA derivative, poly(3-hydroxybutyrate-*co*-3-hydroxyhexanoate) (PHBH), was easily degraded by the formation of biofilms in seawater and freshwater samples (Morohoshi *et al.*, 2018a, 2018b).

Various environmental factors, such as water temperature, water pressure, and ocean currents, affect the degradation of biodegradable plastics that spill into the ocean. One of these factors, marine sand, is expected to have a significant impact on biodegradation because it comes into contact with the surface of plastic when it washes up on the shoreline or sinks to the seafloor. A previous study showed various bacteria attached to the surfaces of marine sand grains (Meadows and Anderson, 1966). Although bacteria may affect the degradation of biodegradable plastics, the effects of marine sand on biodegradable plastic degradation and the microbial communities of biofilms that form on plastic surfaces remain unclear. Therefore, the present study investigated the effects of the addition of marine sand on the degradation of representative biodegradable plastics in seawater samples as well as the bacterial flora of the biofilms that formed on the surfaces of biodegradable plastics.

Seawater and marine sand samples were collected at 36°23′13″N/140°36′58″E on the Ajigaura coast (Hitachinaka, Ibaraki, Japan) on November 23, 2020 (sample S1), March 21, 2021 (sample S2), and April 25, 2021 (sample S3). Cream-colored marine sand was kept moist to prevent dryness and used in experiments on the day of collection. Seawater samples were mixed with 0.5‍ ‍g‍ ‍L^–1^ NH_4_Cl as a nitrogen source and 0.1‍ ‍g‍ ‍L^–1^ KH_2_PO_4_ as a phosphorus source, and were then poured into a 100-mL screw-cap glass bottle in the presence or absence of 30‍ ‍g of marine sand. To assess biodegradability in seawater, four types of general biodegradable plastic films, PLA, PBAT, PBSA, and PHBH, were used. All films had a thickness of approximately 100‍ ‍μm and were prepared by T-die cast extrusion. Square plastic films with dimensions of 1.5×1.5‍ ‍cm were cut from plastic sheets and their initial weight was measured using a precision electronic scale. Plastic films were soaked in seawater samples and then incubated at 25°C with gentle shaking at 150‍ ‍rpm. To maintain aerobic conditions, the screw cap was loosened during the incubation. After plastic films had been incubated for 20 days, they were air-dried and their final weights were measured as described above. All experiments were performed in triplicate. Test results are shown in Fig. 1. No significant weight changes were observed in PLA, PBAT, or PBSA films after the incubation for 20 days in the absence of marine sand. In contrast, the PHBH film exhibited slight weight loss (approximately 30%) in the seawater sample in the absence of marine sand and marked weight loss (approximately 80%) in its presence. This result demonstrated that the addition of marine sand to seawater samples accelerated the degradation of the PHBH film. However, this acceleration of weight loss in the PLA, PBAT, and PBSA films was not detected in the presence of marine sand. Based on these results, the addition of marine sand did not add new degrading activity to the seawater sample, it only accelerated the degradation of specific plastic films that were already degradable in seawater samples in the absence of marine sand.

We previously demonstrated that PHBH was degraded by the formation of biofilms on its surface in seawater samples (Morohoshi *et al.*, 2018a). In the present study, the surfaces of the PHBH film were covered by some biofilms in the seawater sample without marine sand after the incubation for 20 days; however, they were covered with numerous biofilms in the seawater sample with marine sand, with excess biofilms detaching from the PHBH surface and free-floating in the seawater (Fig. 2A). To isolate PHBH-degrading bacteria from the excess biofilms that formed on PHBH films, biofilm suspensions were serially diluted with distilled water, spread on Marine Broth 2216 (MB; Difco) agar plates, and incubated at 30°C for 72 h. Bacterial colonies from each sample were randomly selected and inoculated at the center of MB agar plates containing 1‍ ‍g‍ ‍L^–1^ PHBH powder (Kaneka Corporation) to detect PHBH-degrading activity. After an incubation at 30°C for 1‍ ‍week, the development of clear zones around the colonies, representing PHBH degradation, was evaluated. Five colonies showed clear zones (Fig. 2B). To identify the bacterial species, the genome was extracted from bacterial colonies and the 16S rRNA gene was amplified from total DNA by PCR with Blend Taq-Plus DNA polymerase (Toyobo) using the previously described primers 27f (5′-AGAGTTTGATCMTGGCTCAG-3′) and 1525r (5′-AGGAGGTGWTCCARCC-3′) (Ochiai *et al.*, 2013). PCR was performed using the following cycling parameters: at 94°C for 30‍ ‍s, 50°C for 30‍ ‍s, and 74°C for 1‍ ‍min for 27 cycles. Sequencing was conducted using BigDye Terminator ver. 3.1 and an Applied Biosystems 3500 Series Genetic Analyzer (Applied Biosystems). The closest type-strain 16S rRNA gene relatives of each clone sequence were identified using the Ribosomal Database Project sequence match tool (Cole *et al.*, 2005). The 16S rRNA gene sequences of strains S5 and S10 showed high identity (>99%) with those of *Alteromonas oceani* S35^T^ (DDBJ/ENA/GenBank accession no. MF687202). The 16S rRNA gene sequences of strains S6 and S9 showed high identity (>99%) with that of *Shewanella waksmanii* KMM 3823^T^ (AY170366). The genera *Alteromonas* and *Shewanella* are classified into the family *Alteromonadaceae*. We previously reported that bacterial strains belonging to the family *Alteromonadaceae* functioned as principal PHBH-degrading bacteria in the biofilms that formed on the surfaces of PHBH films in seawater samples (Morohoshi *et al.*, 2018a). The 16S rRNA sequence of strain S7 showed high identity (98.7%) with *Pseudophaeobacter arcticus* DSM 23566^T^ (DQ514304). Although the genus *Pseudophaeobacter* was not identified as PHA-degrading bacteria in the present study, we demonstrated that the family *Rhodobacteraceae*, which includes the genus *Pseudophaeobacter*, may function as low-mole­cular-weight PHBH-degrading bacteria in seawater samples (Morohoshi *et al.*, 2018a). These findings suggest that the composition and function of biofilm-forming bacteria on the surfaces of PHBH films in seawater samples in the presence of marine sand are similar to those of bacteria on the surfaces of PHBH films in seawater samples without marine sand.

The structure of the bacterial community that formed on PHBH films in the seawater sample with or without marine sand was analyzed by a 16S rRNA gene-based metagenomic ana­lysis using MiSeq technology (Illumina). Biofilms were unglued from the surfaces of PHBH films by vortexing and collected by centrifugation at 12,000×*g* for 5‍ ‍min. Total DNA was extracted from the collected biofilm samples using the ISOSPIN Fecal DNA kit (Nippon Gene) and diluted to 10‍ ‍ng μL^–1^. The V1 and V3 regions of the 16S rRNA gene were amplified using V1 (27F) forward and V3 (534R) reverse primer pairs with Illumina overhang sequences (Zheng *et al.*, 2015). Index tag sequences were added to the amplicons using the Nextera XT Index kit (Illumina) and sequenced with the MiSeq Reagent Kit v3 (Illumina). Raw paired-end reads were processed using QIIME 2 version 2021.2.0 (Bolyen *et al.*, 2019). Paired-end reads were trimmed and merged, and chimeric sequences were removed using the DADA2 plugin in QIIME 2 (Callahan *et al.*, 2016). Operational taxonomic units (OTUs) were clustered using DADA2 and classified by the QIIME2 pre-trained naïve Bayes classifier trained on Silva 138 99% OTU full-length sequences (Quast *et al.*, 2013).

The family *Rhodobacteraceae*, which was predicted to be PHBH-degrading bacteria, was observed in all biofilm samples as the predominant family (Fig. 3), followed by *Rhizobiaceae*, *Terasakiellaceae*, *Acrobacteraceae*, and *Phicisphaeraceae*, which differed among experimental replications and in the presence or absence of marine sand (Fig. 3). However, there is currently no evidence to support bacterial isolates belonging to these families exhibiting PHA-degrading activity. Therefore, these families are assumed to use 3-hydroxyalkanoate as a carbon source released from PHBH films degraded by the family *Rhodobacteraceae*. To identify the bacterial species that predominantly changed in the presence or absence of marine sand, differentially abundant taxa in each group were identified via an ana­lysis of microbiome composition (ANCOM) (Mandal *et al.*, 2015). ANCOM results showed that the genus *Dethiosulfatibacter*, which belongs to the family *Dethiosulfatibacteraceae*, was significantly abundant in seawater samples with marine sand (Fig. S1). However, a bacterial isolate belonging to the genus *Dethiosulfatibacter* that exhibits PHA-degrading activity has yet to be identified. *Dethiosulfatibacter* is an anaerobic bacterium with sulfate-reducing activity (Takii *et al.*, 2007). In our experiments, black pigmented marine sand was noted under the biofilm-forming PHBH film after the 20-days incubation at 25°C (Fig. 2A). This black pigmentation suggests that the boundary between marine sand and the PHBH film was an anaerobic condition, and black-colored sulfide was produced due to sulfate reduction by bacteria in the *Dethiosulfatibacteraceae* family. Although the family *Alteromonadaceae* was mainly isolated as PHBH-degrading bacteria from the biofilms that formed on the surfaces of PHBH films in the present study, a metagenomic ana­lysis revealed that the family *Alteromonadaceae* was not abundant in the bacterial community of biofilms. We previously demonstrated that the family *Alteromonadaceae* was the principal PHBH-degrading bacterial family in the early stages of biofilm formation, but almost disappeared from biofilms in the late stages when the degradation of plastic had markedly progressed (Morohoshi *et al.*, 2018a). These findings assumed that bacteria in the family *Alteromonadaceae* were easily isolated on MB agar medium, but did not really contribute to the degradation of PHBH films in the late stages when we collected samples because of their low abundance.

To investigate the effects of marine sand on the composition of bacterial communities in biofilms in more detail, beta diversity was measured by a principal coordinate ana­lysis (PCoA) plot based on unweighted and weighted UniFrac distances (Lozupone *et al.*, 2007). PCoA plots are shown in Fig. S2. Although PCoA results revealed that the bacterial community structure of biofilms in seawater with marine sand was separate from that of biofilms in seawater without marine sand, the effects of the addition of sand to the seawater sample on the microbial community structure was not clear. Therefore, to examine relationships among microbiota in biofilms, a pairwise permutational multivariate ana­lysis of variance (PERMANOVA) (Anderson, 2001) was used, and *P*-values were produced with 999 permutation tests. However, the results of PERMANOVA showed no significant difference among samples in the presence or absence of marine sand (Tables S1 and S2).

In conclusion, we herein investigated the effects of the addition of marine sand on the degradation of biodegradable plastics. Among the four types of biodegradable plastics examined in the present study, only PHBH was degraded in seawater samples, and its degradation was markedly accelerated in the presence of marine sand. On the other hand, the addition of marine sand significantly increased the amount of biofilms that formed on the surfaces of PHBH films, but did not affect their bacterial composition from that of biofilms in the absence of marine sand. Although the family *Rhodobacteraceae* is considered to be the principal PHBH-degrading bacterium and accounts for a large proportion of the bacterial communities in biofilms, its composition in biofilms did not significantly differ between the presence and absence of marine sand. Based on these results, the addition of marine sand did not significantly change the composition of bacteria in biofilms, but may have served as a source of bacteria involved in the formation of biofilms and degradation of PHBH, resulting in enhanced film degradation.

## Nucleotide sequence accession number

The 16S rRNA gene sequences from PHBH-degrading isolates were deposited in the DDBJ/ENA/GenBank database under accession numbers LC716641 to LC716645. Raw Illumina MiSeq sequencing data were deposited in the DDBJ Read Archive (DRA) under the accession number DRA014363.

## Citation

Morohoshi, T., Taniguchi, A., Sugawara, A., Suzuki, T., and Sato, S. (2022) Effects of Marine Sand on the Microbial Degradation of Biodegradable Plastics in Seawater and Biofilm Communities that Formed on Plastic Surfaces. *Microbes Environ ***37**: ME22047.

https://doi.org/10.1264/jsme2.ME22047

## Supplementary Material

Supplementary Material

## Figures and Tables

**Fig. 1.**
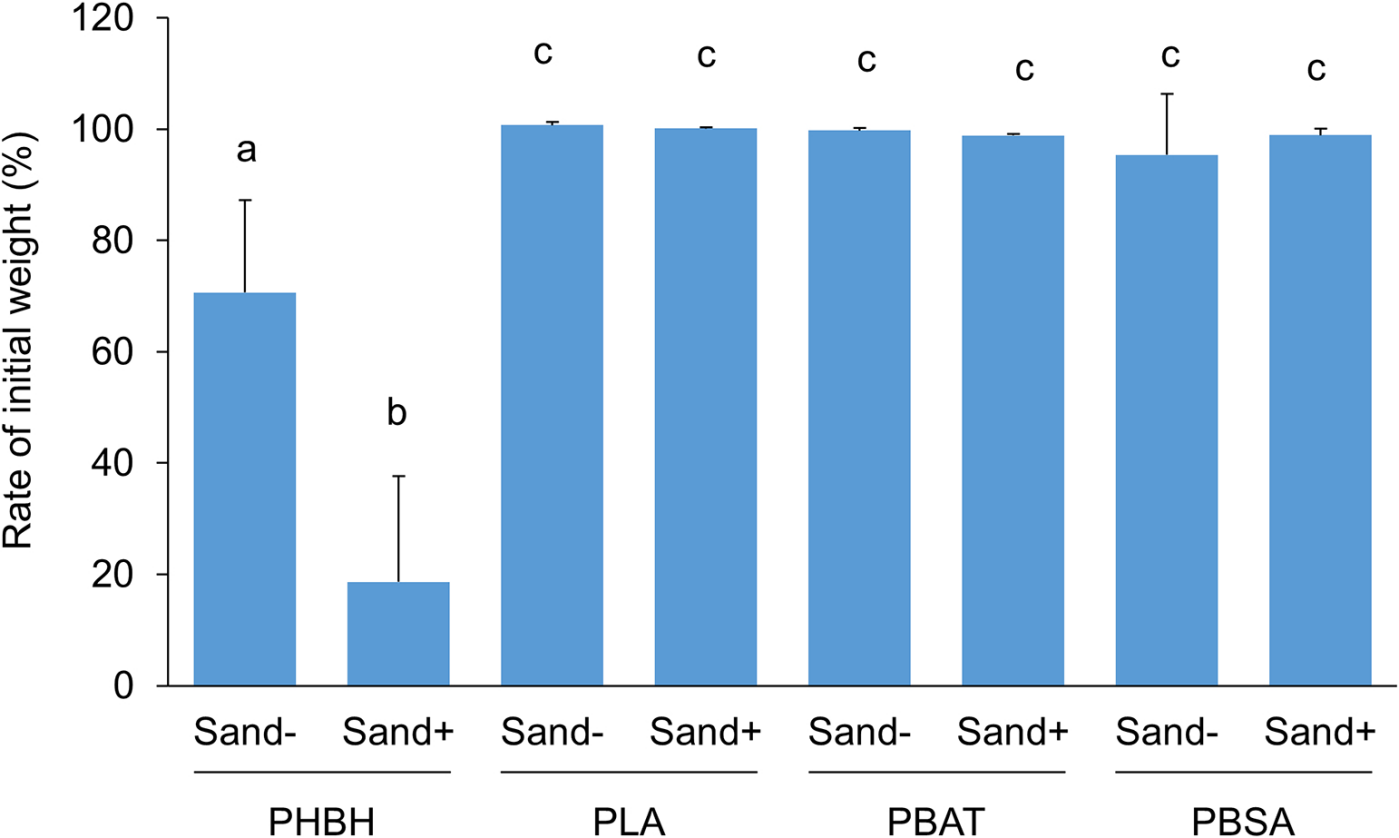
Percentage of the final weight relative to the initial weight of biodegradable plastics (PHBH, PLA, PBAT, and PBSA) incubated at 25°C for 20 days in seawater samples in the presence or absence of marine sand. The results obtained were reproduced in at least three independent samples, and error bars indicate standard deviations. Different lowercase letters indicate significant differences by Tukey’s HSD test (*P*<0.05).

**Fig. 2.**
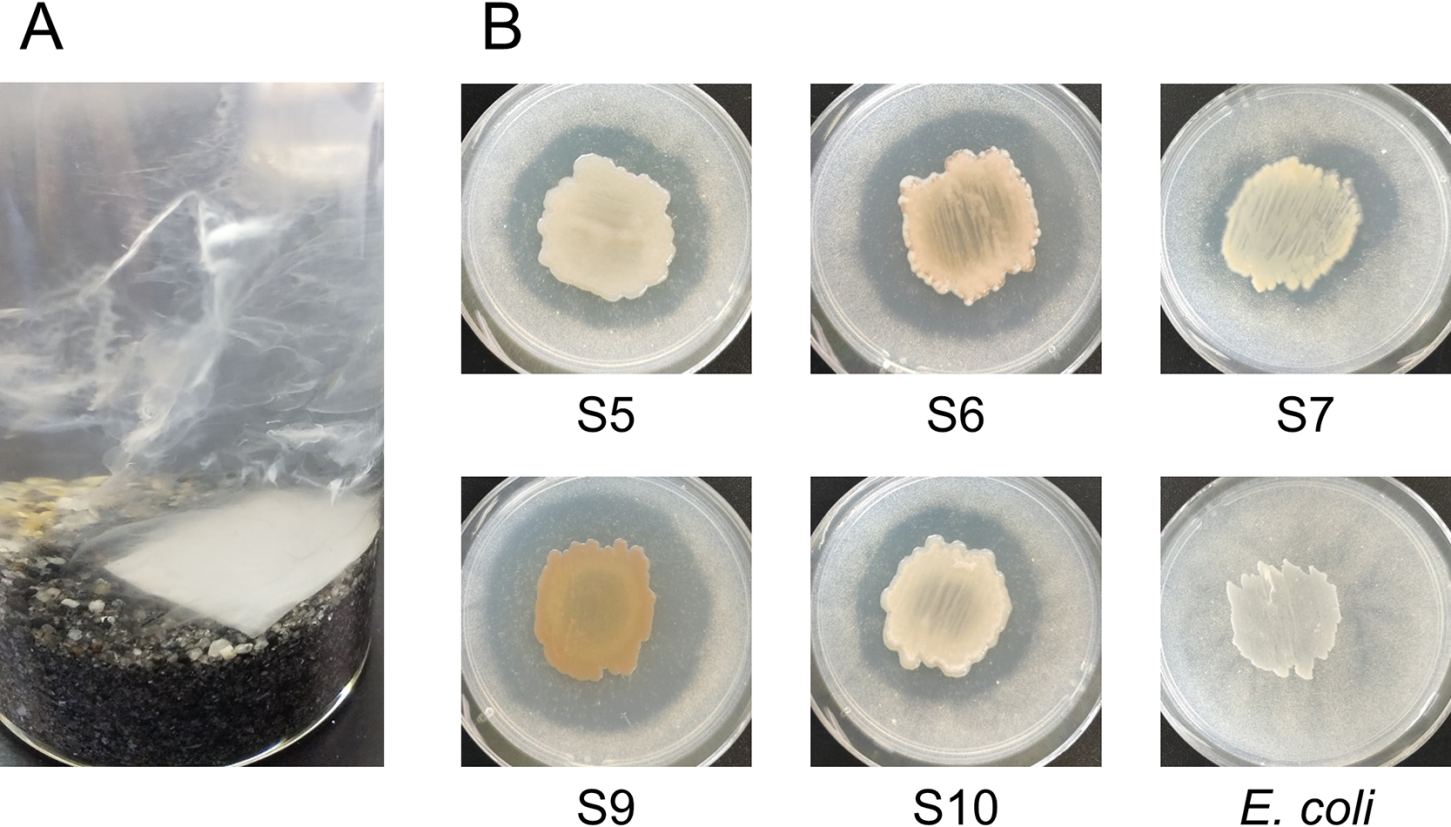
(A) Biofilms on PHBH films incubated at 25°C for 20 days in seawater samples in the presence of marine sand. Excess biofilms detached from the PHBH surface were free-floating in the seawater, with the appearance of a white haze. (B) PHBH-degrading activity of bacterial isolates. PHBH-degrading strains (S5, S6, S7, S9, and S10) and the negative control strain (*E. coli*). PHBH-degrading activity was detected on the MB-PHBH agar plate after an incubation at 30°C for 1‍ ‍week. The development of clear zones around the colonies indicated the degradation of PHBH.

**Fig. 3.**
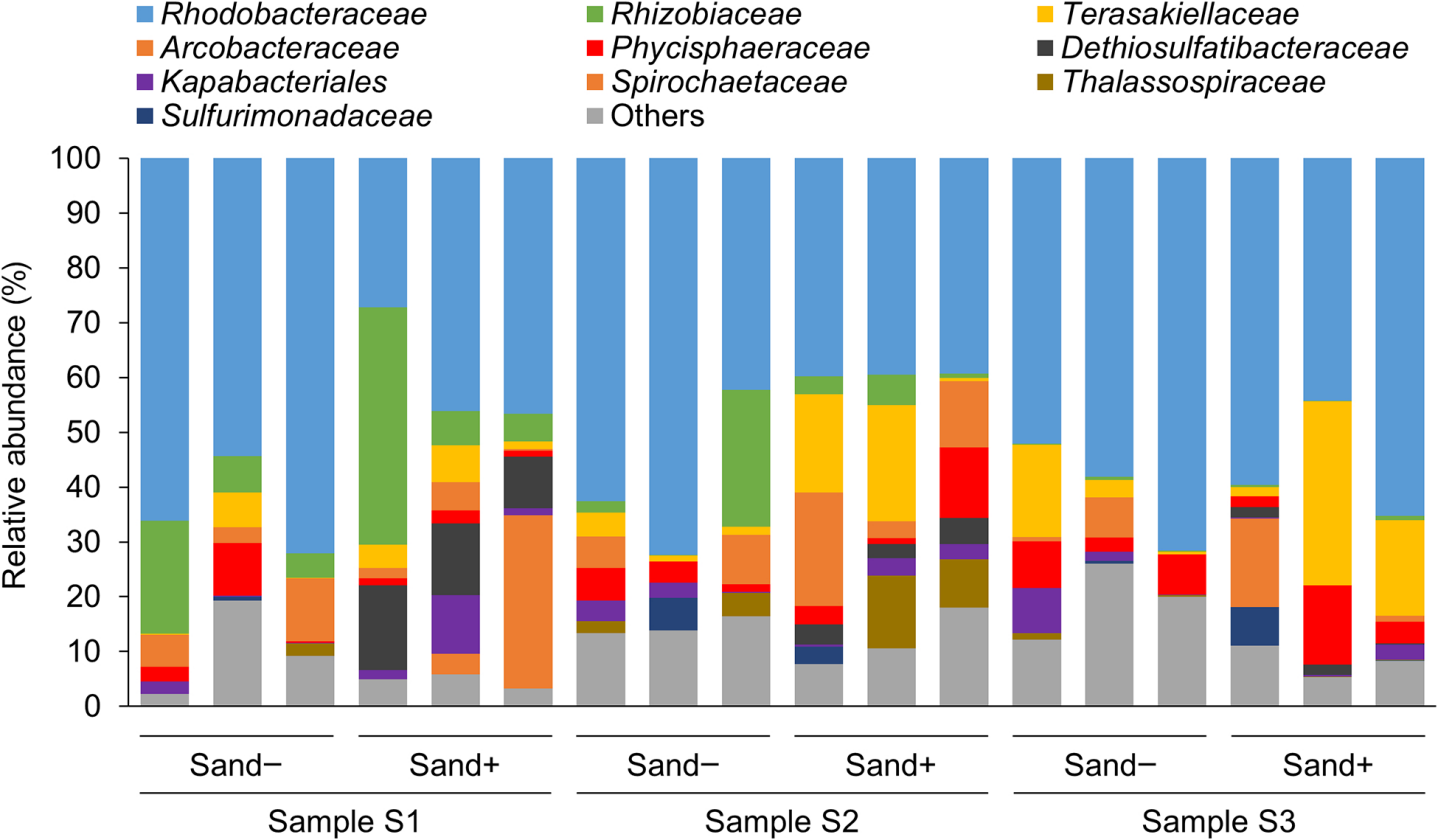
Relative abundance of top ten groups classified at the family level in biofilms that formed on PHBH films in seawater samples in the presence or absence of marine sand.
