# Supplementary material for: Effects of Marine Sand on the Microbial Degradation of Biodegradable Plastics in Seawater and Biofilm Communities that Formed on Plastic Surfaces
Source: Microbes Environ. 2022 Oct 15;37(4):ME22047. doi: 10.1264/jsme2.ME22047 (PMC9763043; doi:10.1264/jsme2.ME22047)
Supplement: Supplementary file 1 — Supplementary Material [file 37_22047_s1.pdf]

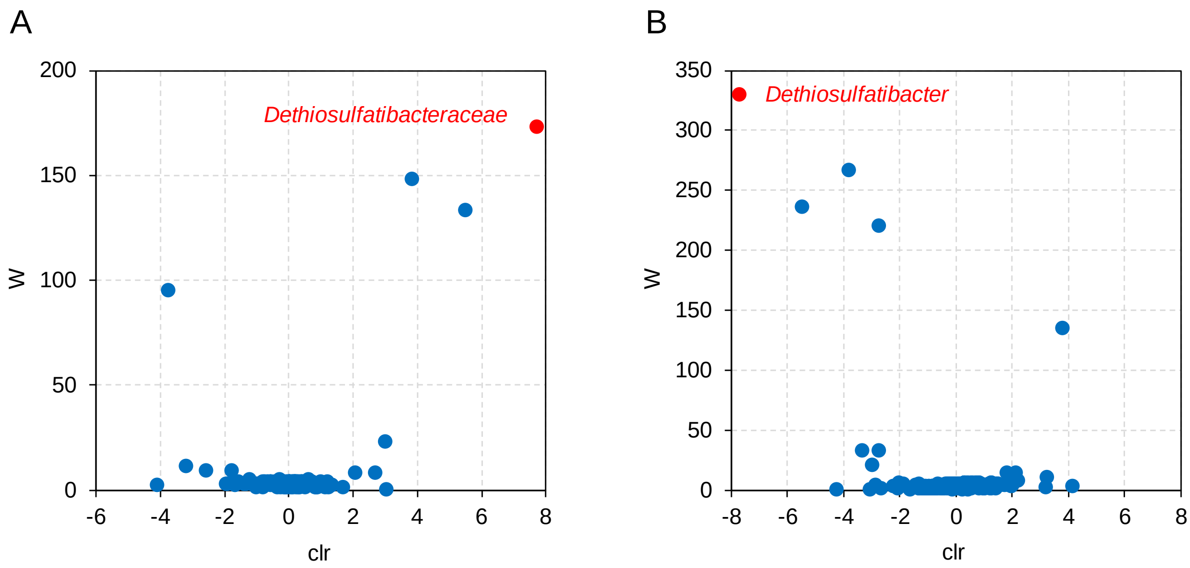

Figure S1. Differential microbiota abundance in biofilms formed on PHBH films in seawater samples in the presence or absence of marine sand. Volcano plot for the analysis of microbiome composition (ANCOM) test for family (A) and genus (B) levels. Only significant bacterial taxa are labelled and colored in red. Significant taxa showed high w-statistics.

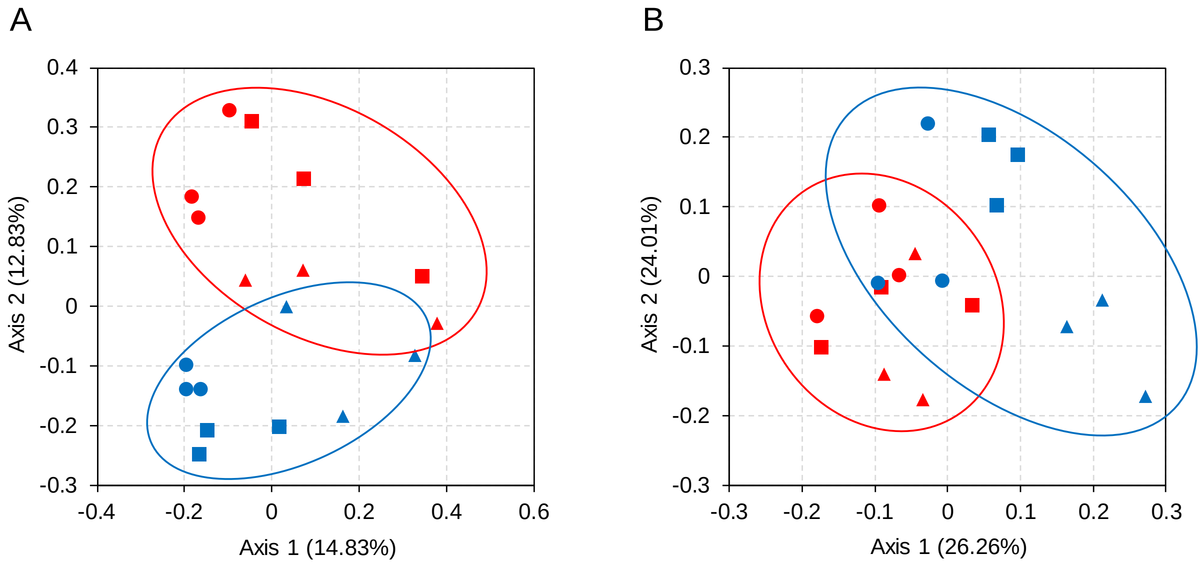

Figure S2. Principal coordinate analysis (PCoA) plots were generated based on the calculated distances in the unweighted (A) and weighted (B) UniFrac matrices. The blue and red colors represent the biofilm samples in seawater with and without marine sand, respectively. The sampling date is shown in squares (sample S1), circles (sample S2), and triangles (sample S3).

Table S1. Pairwise PERMANOVA results based on the unweighted UniFrac distance matrix.

Group 1	Group 2	pseudo-F	p-value	q-value
S1	S1 +Sand	0.784570	0.898	0.8980
	S2	1.130477	0.515	0.5518
	S2 +Sand	1.620727	0.080	0.1425
	S3	1.346223	0.101	0.1425
	S3 +Sand	1.803514	0.095	0.1425
S1 +Sand	S2	1.169646	0.213	0.2458
	S2 +Sand	1.469345	0.112	0.1425
	S3	1.957663	0.081	0.1425
	S3 +Sand	1.781200	0.107	0.1425
S2	S2 +Sand	1.923291	0.114	0.1425
	S3	1.404401	0.096	0.1425
	S3 +Sand	2.206256	0.110	0.1425
S2 +Sand	S3	2.386992	0.102	0.1425
	S3 +Sand	1.583555	0.105	0.1425
S3	S3 +Sand	1.937719	0.098	0.1425

Table S2. Pairwise PERMANOVA results based on the weighted UniFrac distance matrix.

Group 1	Group 2	pseudo-F	p-value	q-value
S1	S1 +Sand	2.715034	0.104	0.2138
	S2	0.631247	0.890	0.8900
	S2 +Sand	4.129520	0.114	0.2138
	S3	1.743663	0.303	0.4132
	S3 +Sand	1.826769	0.292	0.4132
S1 +Sand	S2	2.808036	0.106	0.2138
	S2 +Sand	3.159132	0.112	0.2138
	S3	4.022585	0.114	0.2138
	S3 +Sand	2.722095	0.101	0.2138
S2	S2 +Sand	2.939600	0.086	0.2138
	S3	0.796127	0.691	0.7404
	S3 +Sand	1.073289	0.389	0.4863
S2 +Sand	S3	3.973194	0.094	0.2138
	S3 +Sand	1.449343	0.194	0.3233
S3	S3 +Sand	0.928459	0.586	0.6762
